# Content-Based Image Retrieval for Traditional Indonesian Woven Fabric Images Using a Modified Convolutional Neural Network Method

**DOI:** 10.3390/jimaging9080165

**Published:** 2023-08-18

**Authors:** Silvester Tena, Rudy Hartanto, Igi Ardiyanto

**Affiliations:** 1 Department of Electrical Engineering and Information Technology, Universitas Gadjah Mada, Yogyakarta 55281, Indonesia; siltena@mail.ugm.ac.id (S.T.); igi@ugm.ac.id (I.A.); 2 Department of Electrical Engineering, University of Nusa Cendana, Kupang 85001, Indonesia

**Keywords:** ikat woven fabric, image retrieval, pretrained CNN, modified CNN

## Abstract

A content-based image retrieval system, as an Indonesian traditional woven fabric knowledge base, can be useful for artisans and trade promotions. However, creating an effective and efficient retrieval system is difficult due to the lack of an Indonesian traditional woven fabric dataset, and unique characteristics are not considered simultaneously. One type of traditional Indonesian fabric is ikat woven fabric. Thus, this study collected images of this traditional Indonesian woven fabric to create the TenunIkatNet dataset. The dataset consists of 120 classes and 4800 images. The images were captured perpendicularly, and the ikat woven fabrics were placed on different backgrounds, hung, and worn on the body, according to the utilization patterns. The feature extraction method using a modified convolutional neural network (MCNN) learns the unique features of Indonesian traditional woven fabrics. The experimental results show that the modified CNN model outperforms other pretrained CNN models (i.e., ResNet101, VGG16, DenseNet201, InceptionV3, MobileNetV2, Xception, and InceptionResNetV2) in top-5, top-10, top-20, and top-50 accuracies with scores of 99.96%, 99.88%, 99.50%, and 97.60%, respectively.

## 1. Introduction

Traditional Indonesian fabrics have charming patterns that represent a variety of unique features. Each region in Indonesia has traditional fabrics that are based on the lives of the local people. Local wisdom based on each culture must be cultivated and preserved to prevent extinction. The Indonesian government is persistently providing various local cultural products, including traditional fabrics, to UNESCO (the United Nations Educational, Scientific, and Cultural Organization) for recognition at the WTO (World Trade Organization). Ikat woven fabrics are valuable cultural assets of Indonesia as they are used in all common occasions, such as birth, wedding, and death ceremonies. The ikat woven fabrics of East Nusa Tenggara also have unique traditional motifs that are in line with the local culture. The ikat woven fabrics have unique color, texture, and shape characteristics. Each area has a different motif that matches the culture of the local community. In addition, they include patterns with different animals, flowers, and geometric shapes [1]. Each region has a different motif, so it is difficult to identify the type and area of origin. An effective electronic recognition system is needed to identify the type of ikat woven fabric. Content-based image retrieval (CBIR) is a technique used to identify the type of ikat woven fabric. The ikat woven fabric retrieval system can be used by the educational world to study local content. In addition, it contains a knowledge base for ikat woven fabric artisans and trade promotion individuals at national and international levels.

The most important tasks in image retrieval are feature extraction and similarity measurement. A good feature extraction method affects the recognition performance of an object. The database images are graded in descending order according to their similarity to the search images. Therefore, any image retrieval system’s performance depends on the image similarity measurement. The method used to calculate the similarity measure between two images should ideally be discriminative, reliable, and efficient [2]. In various reports, convolutional neural network (CNN) methods have been used to extract features to overcome semantic gaps and manage large datasets. CNNs are widely used for image recognition, pattern recognition, and speech recognition. Color, texture, shape, and spatial information can be used as features to create an algorithm [3,4,5]. Several image retrieval studies have been conducted on various datasets using pretrained CNN models with transfer learning methods. In addition, extensive research has been conducted on transfer learning in models as a common technique in image processing, recognition, and classification tasks [6], especially when training data are lacking. Research on the retrieval of ikat woven fabrics has been carried out by several researchers using handcrafted features [6,7]. However, the researchers used a small number of ikat woven fabric images and limited features.

Based on the image retrieval process, pretrained CNNs have been used to extract features in various reports to overcome the semantic gap and manage large datasets [3,5,8]. A pretrained CNN model has been reported to provide a higher retrieval accuracy than handcrafted feature extraction techniques [9,10]. The features learned from a pretrained CNN model can effectively serve as a generic model of the visual world. Therefore, the model can be used for various computer vision problems. The transfer learning method is a better solution for image recognition compared to training a network with millions of parameters or developing new paradigms from scratch [11]. Several research works have recently applied transfer learning to some pretrained CNNs, such as VGG [12,13,14], Res-Net [15], GoogLeNet, Inception [16], MobileNet [17], AlexNet [18,19,20], and DenseNet [21]. Fine-tuning a pretrained model for a new task is an efficient transfer method to fill the various knowledge transfer gaps for CNN models [22]. In the standard transfer learning example, a model is trained with a large quantity of data and learns model parameter weights. The model is then integrated with a new model for the target task, which can be learned with pretrained weights and fine-tuned on the target dataset [23]. Previous research has used transfer learning for ikat woven fabric image retrieval, but the top-5 and above accuracy is still low [24]. Research on the retrieval of ikat woven fabrics has been carried out by several researchers using handcrafted features. However, the number of datasets is limited, and the accuracy is below 90% [6,7]. For feature indexing and binary conversion, developing hashing algorithms also increases query time due to the generation of numerous images [15]. In the similarity measurement model, the utilized methods were the Euclidean and Hamming distances, cosine similarity, and Manhattan [8,9,10,25]. 

However, for images with many complex patterns and textures, the image retrieval accuracy of complex patterns and textures is lower than that for images with numerous colors [8,22,23,25,26]. In addition, there are large feature dimensions [27,28] and high computation times [29,30]. This study built a fabric dataset with complex patterns and textures. Furthermore, CBIR is designed with a modified CNN according to the characteristics of the dataset to increase retrieval accuracy. The proposed CBIR model will be useful for ikat woven fabric artisans and trade promotions.

In this study, the advantages of the CNN method for feature extraction are used and adapted to the dataset’s characteristics. This research was conducted in several steps: (1) the pretrained CNN model was evaluated and fine-tuned on new datasets, (2) a modified CNN architecture was used as a feature extractor, and (3) the training and retrieval processes of both models were evaluated. The contributions of this study can be described as follows:

1. A new dataset, namely TenunIkatNet, was used to test several pretrained CNN models to determine their image retrieval performance.
2. A modified CNN architecture model that fits the image characteristics of ikat woven fabrics was created.

The rest of the paper is divided into various parts, including Section 2, which explains the materials and describes the proposed methods. Section 3 describes the research results and discussion. Finally, Section 4 concludes the paper.

## 2. Materials and Methods

### 2.1. TenunIkatNet Dataset

**Figure 1:**
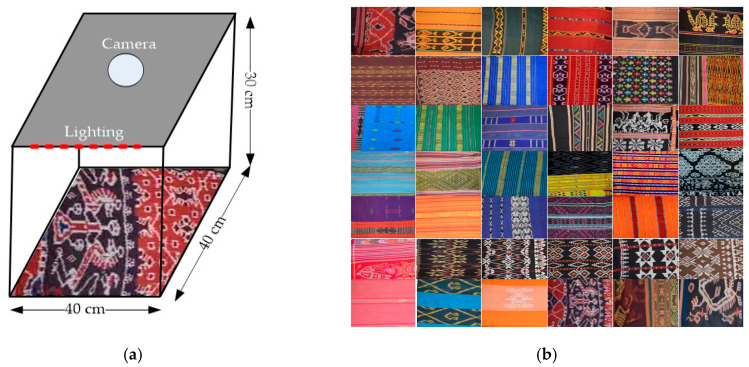
TenunIkatNet dataset. (**a**) The process of capturing images with a mini studio box; (**b**) image samples of ikat woven fabric.

The TenunIkatNet dataset was obtained by photographing each fabric type at several local fabric stores and artisan shops. This dataset consists of 120 types of ikat woven fabrics with different motifs from 17 regions in East Nusa Tenggara, Indonesia. The total dataset contains 4800 images, and there are 40 images of each type of ikat woven fabric. The shooting variations for this dataset were based on luminance, and the fabrics were placed on different backgrounds, hung, and worn on the body, according to the utilization patterns. For luminance, the fabrics were placed in a mini studio box with good lighting, as shown in Figure 1. All of the ikat woven fabrics were photographed with a Nikon D-5600 camera. The image size, lens focal length, and aperture were 24 megapixels, 18–55 mm, and 3.5–5.6 G, according to the camera specifications. 

Ten images were internally and externally captured within the ministudio box, and each image acquisition method mentioned above was carried out every five iterations. The variation in image acquisition, namely changes in illumination and geometry, tests the feature extraction method’s ability to recognize the types of ikat woven fabrics. The perspective of a standard dataset consists of photographic equipment, environmental conditions, the viewing angle, the number of captures, and the image size [7,8]. The TenunIkatNet dataset was collected with good-quality camera equipment and under different conditions that affect luminance and viewing angles. Ikat woven fabrics were worn on the body to collect images with wrinkles. To increase the number of data samples, an augmentation process was carried out. In this study, augmentation processes were applied, such as rotation, zooming, flipping, and cropping.

### 2.2. Proposed Framework

**Figure 2:**
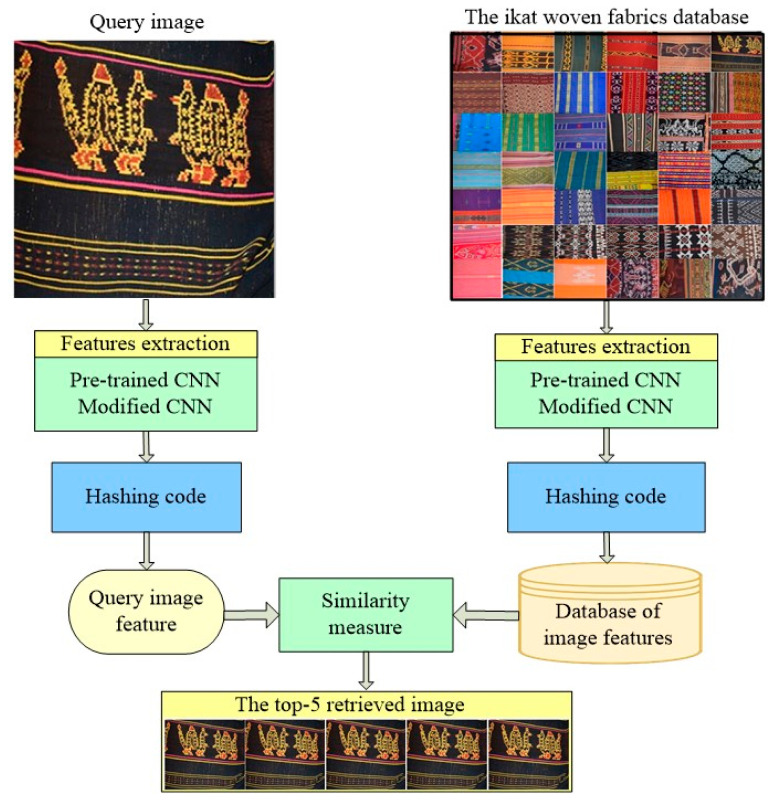
Framework of the content-based ikat woven fabric image retrieval approach.

The workflow of the ikat woven fabric image retrieval system is depicted in Figure 2. The feature extraction process for the image database of ikat fabrics and query images is performed using both pretrained CNN and modified CNN models. The two models are run separately for both training and querying. In this study, the feature vector is converted into binary code to speed up the similarity measurement process [25]. The basic principle is that the extracted feature vectors are grouped based on similarity. Some previous research on image retrieval uses hashing codes to speed up retrieval. One of the most effective hashing methods used is locality-sensitive hashing (LSH) [2,31]. The feature vector of the image is acquired through training the input hashing code using the locality-sensitive hashing (LSH) method. The hashing method is required to speed up image queries. The Hamming distance measures the similarity between the search image and the retrieved images from the database. The retrieved images are displayed in the order of the Hamming distance values with the top-1, top-5, top-10, top-20, and top-50 accuracies. The display results are based on the class number of the ikat woven fabric images.

### 2.3. Pretrained CNN Model

Transfer learning was used to retrieve images of ikat woven fabrics due to the lack of training data. The CNN model is divided into two sections: the convolution layer (CL) at the front and the fully connected layer (FCL) at the back. Here, the CL was used for image feature extraction, while the FCL was used for feature classification [32]. After the last CL, the classifier comprises two commonly used components: the FCL and global average pooling (GAP) [33]. Experiments determined that GAP performed the best; hence, it was selected for feature extraction. Each feature map after the final CL was aggregated and sent directly to the softmax layer to prevent overfitting and enhance the model’s generalizability. For CNN-based image retrieval, features are taken from the pretrained CNN method for image classification using two primary components: the attributes extracted from the CL and the output features from the FCL.

**Figure 3:**
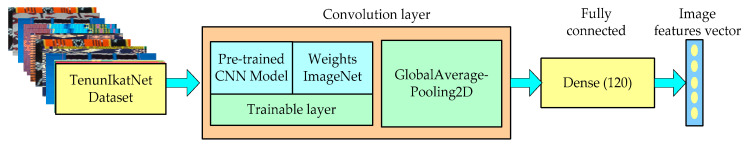
Feature extraction based on a pretrained CNN model.

In this study, several pretrained CNN models were used as feature extractors. The pretrained models include ResNet101, VGG16, DenseNet201, InceptionV3, MobileNetV2, Xception, and InceptionResNetV2. A fine-tuning process was performed to improve the retrieval accuracy and weight adjustment of ImageNet on the ikat woven dataset, as shown in Figure 3.

1. ResNet101

The residual network is an extended version of VGGNet with smaller filters and less sophistication. The CL has a 3 × 3 filter size, and ResNet101 has 100 convolution kernels from Conv1 to Conv5. Immediately following Conv1 with stride 2, downsampling is applied. In the first convolution layer, the kernel size is 7 × 7. Network termination consists of a GAP layer and a 1000-way FCL with softmax [34].

2. VGG16

The VGG16 architecture is a great model with deeper layers and small convolution filter sizes for large datasets. The model involves thirteen convolutional layers followed by an *ReLU* layer and three FCLs. The convolution and MP filter dimensions are 3 × 3 and 2 × 2, respectively. This model’s advantage is its highly homogeneous architecture, which performs only 3 × 3 convolutional and 2 × 2 pooling operations end to end. The drawbacks of VGG16 are that its results are more difficult to evaluate, it requires more memory, and it has 138 million parameters [35].

3. DenseNet201

The DenseNet201 architecture consists of four dense blocks and three transition layers, each of which has a dense block process involving batch normalization, *ReLU* activation, and convolution with a 3 × 3 filter. The layer between two neighboring blocks is called the transition layer, and the feature size changes throughout convolution and average pooling [21].

4. InceptionV3

The InceptionV3 architecture was designed to identify the best local sparsity configuration in a convolutional vision network, resulting in improved performance with remarkably less computation. Generally, the InceptionV3 network consists of modules similar to those mentioned above stacked upon one another. In addition, a stride of two is utilized for MP. The output is compiled and forwarded to the subsequent inception module. Before 3 × 3 and 5 × 5 convolution, 1 × 1 kernel convolution is applied to limit the number of input channels. The 1 × 1 convolution process is significantly less expensive than the 5 × 5 process and is easily circumvented by decreasing the number of input channels. InceptionV3 consists of 103 CLs, four MP layers, and five average pooling layers [36].

5. MobileNetV2

MobileNetV2 is a CNN architecture with an efficient model size and a small capacity of 14 MB. The MobileNetV2 model includes a full convolution layer with 32 filters and 19 bottleneck layers. MobileNetV2 employs depthwise separable convolutions to build compact deep neural networks. MobileNetV2 uses width and resolution multipliers to reduce size and latency by sacrificing a significant amount of accuracy [37].

6. Xception

The Xception architecture is slightly superior to InceptionV3 for the ImageNet dataset and significantly outperforms InceptionV3 for image classification with a 350 million-image, 17,000-class dataset. Xception has the same parameters as InceptionV3 but is superior in terms of efficiency [38].

7. InceptionResNetV2

InceptionResNetV2 is a CNN architecture derived from the Inception family of architectures but with residual connections. InceptionResNetV2 comprises three modules designated A, B, and C. InceptionResNetV2 introduces “reduction blocks” that alter the grid’s width and height. This model consists of 449 layers [36].

### 2.4. Proposed Modified CNN Architecture

The CNN modification proposed in this study is adapted to the image characteristics of ikat woven fabric. Ikat woven fabrics, which are rich in color, texture, and shape features, require appropriate feature extraction methods. The proposed method can preserve the features of ikat woven fabric in the training phase. The obtained feature vector is utilized during the retrieval process, preceded by hashing code. The objective of designing a specific CNN architecture for ikat woven fabric images is to improve the retrieval accuracy, reduce the computational load, and decrease the model size and retrieval time. In addition, the CNN architecture is based on the number of TenunIkatNet dataset categories. Several pretrained models have been evaluated for ikat woven fabric image retrieval without treatment with suboptimal results [24]. The pretrained models were trained on significantly different image datasets with different classes. In the TenunIkatNet dataset, illumination and image geometry are altered, resulting in the development of a unique CNN architecture.

**Figure 4:**
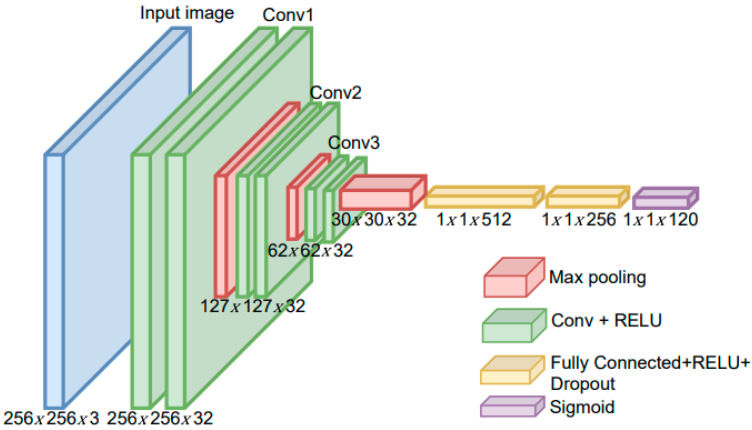
Proposed modified CNN architecture.

The modified CNN model depicted in Figure 4 is utilized to directly extract image features from the input image and classify the ikat woven fabric image into 120 classes. The modified model uses a random search hyperparameter tuning strategy to obtain the best performance. The random search method consumes less time and resources [39]. The architecture consists of three convolution layers, max-pooling, and FCLs. 

The following section explains the modified CNN architecture in Figure 4 in more detail.

#### 2.4.1. Convolution and Max-Pooling Layer

**Figure 5:**
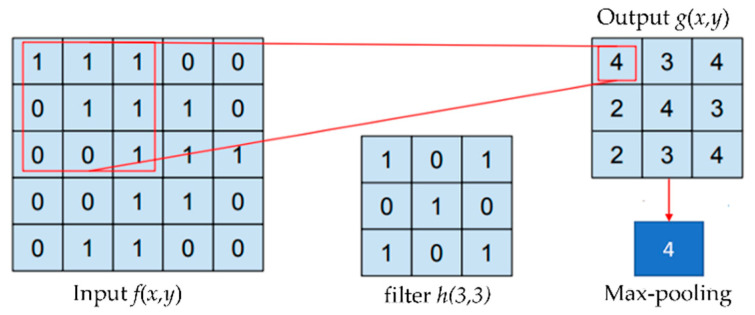
Illustration of the convolution and max-pooling processes.

The input and filter image convolution process can be formulated as follows:

*g*(*x*,*y*) = *f*(*x*,*y*) *xh*(*m*,*n*) (1)

where *h*(*m*,*n*) represents the filter, *g*(*x*,*y*) is the resulting convolution image, and *f*(*x*,*y*) is the original image. An illustration of the convolution process between input images and the filter based on Equation (1) is shown in Figure 5. The three convolution layers are quite effective at extracting the features of ikat woven fabrics. A shallower layer is selected because the image of the ikat woven fabric contains many geometric features. These features are extracted by the initial convolution layer. Each convolution layer applies 32 filters to obtain the map features. In this research, the input image size is 256. The filter size is 3 × 3 pixels, and the stride value used with the pooling layer is 2. A small filter size provides the best performance [40] and less computational load [41]. In general, the stride value should be less than twice the filter size [41,42]. Along the spatial dimensions, a pooling layer is applied for downsampling.

#### 2.4.2. Fully Connected Layer

In the fully connected layer, dense and dropout layers are added. The number of nodes in each dense layer decreases as the process moves toward the output layer. In this study, two dense layers were placed after the flattening process, and the probability value used for evaluating the modified CNN architectures is *p* = 0.2 [43]. The dropout technique prevents overfitting problems due to limited datasets [17,44]. 

#### 2.4.3. Activation Functions

The process of normalizing image feature weights uses the activation function. This study uses a nonlinear activation function, the rectified linear unit (*ReLU*), and softmax. The *ReLU* activation function is applied to the convolution layer and is fully connected. The *ReLU* activation equation is as follows:

(2) $$ReLU=\left\{\begin{array}{c} 0,\;x\leq 0\\ x,\;x>0 \end{array}\right.\;$$

where *x* is the input value. In the output layer, softmax activation is used. The output value of the softmax activation function is between 0 and 1 [45].

(3) $$\sigma {\left(z\right)}_{j}=\frac{e^{z_{j}}}{\sum_{k=1}^{K}e^{z_{k}}}\;\;for\;j=1,\ldots ,\;K$$

where $\sigma {\left(z\right)}_{j}$ is the probability value for the *j*th class, $e^{z_{j}}$ is the output of the *j*th node,$\;e^{z_{k}}$ is the output of each existing node, and *K* is the number of classes. The value of *K* in this study is 120 and represents the number of ikat woven fabric types.

#### 2.4.4. Loss Function

The loss function is a mathematical equation used to calculate the loss value. The loss value is used in the backpropagation process to evaluate parameters such as weights and bias to improve the neural network for optimal performance. In this study, we used categorical cross entropy. Cross entropy measures the difference between two probability distributions for a particular random variable. The entropy equation is as follows:

(4) $$H=-\sum_{x=1}^{N}P\left(x\right)\log\left(P\left(x\right)\right)$$

where *H* is the entropy, *x* is the input data, *N* is the number of data, and *P* is the probability.

### 2.5. Performance Evaluation

Performance is evaluated based on the accuracy and error rate [18,25]. In addition, the F1-score can be used to properly evaluate the performance of the model [12]. The performance of image retrieval is the system’s capacity to obtain images from a database in response to user requests. The total data number represents the number of images shown in the top-*k* results during the search process.

(5) $$Accuracy=\frac{total\;correct\;image}{total\;number\;image\;query}\times 100\%$$

(6) $$E@k=\frac{\sum_{i=1}^{k}R\left(i\right)}{k}$$

where E denotes the error and k represents the number of outputs to be analyzed. The ground truth relevance of both a query image and the i-th ranked image is represented by $R\left(i\right)$. In this research, only the image’s appearance is considered, and $R\left(i\right)\in \left\{0,1\right\}$, where 1 indicates that the search image and the i-th image have different appearances and 0 indicates that the opposite is true.

## 3. Results and Discussion

### 3.1. Experimental Settings

The proposed scheme was developed using the TensorFlow framework for deep learning. This study used Google Collaboratory facilities for all experiments, with the following hardware specifications: Intel (R) Xeon(R) CPU @ 2.20 GHz, GPU NVIDIA-SMI memory of 16 GB, 12 GB RAM, and 358 GB disk space. The Adam method was selected for parameter optimization. Dropout was implemented to prevent overfitting and accelerate the process of learning. In the process of fine-tuning the parameters, the learning rate and batch size were set to 0.001 and 64, respectively. These parameters were applied equally to both groups of pretrained CNN and modified CNN models. This experiment used a 6 bit hashing code to index image features [24]. In this experiment, the datasets are divided into a training set of 80% and a testing set of 20%.

### 3.2. Experimental Results

#### 3.2.1. Training Process Evaluation

Training was carried out for the pretrained CNN model and modified CNN. The pretrained model used in this research was an intelligent model trained on a large dataset, called ImageNet. The training process used fine-tuning strategies for adjusting ImageNet weights for the TenunIkatNet dataset. GAP was applied in the fully connected layer to prevent overfitting, minimize the number of parameters, and decrease the model size.

**Figure 6:**
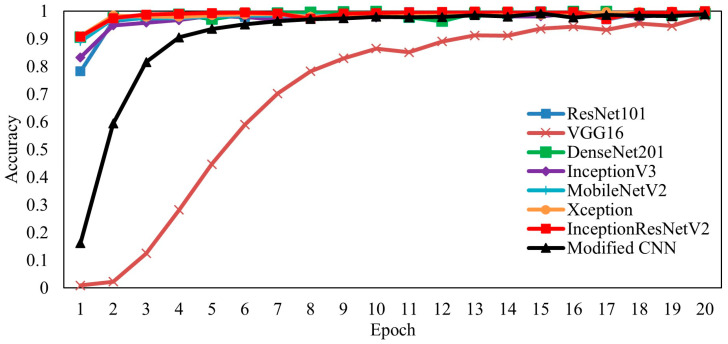
Visualization of training accuracy on pretrained and proposed models.

Figure 6 compares the training accuracy of seven pretrained CNN models and a modified CNN architecture. Based on the training data in the graph, the pretrained ResNet101, DenseNet201, InceptionV3, MobileNetV2, Xception, InceptionResNetV2, and VGG16 models were overfitted. Overfitting can occur when the amount of data is small and the new dataset features are complex. The VGG16 model cannot analyze the relationship between variables in the data or predict or classify new data points. In this circumstance, weight adjustment cannot be performed correctly. Pretrained models are trained for different classification needs and data characteristics. The VGG16 model cannot predict or classify new data points by studying the relationship between variables in ikat woven fabric images. According to the experimental results, the modified CNN model increased the stable training accuracy from the second to the twentieth epoch. In contrast, the pretrained model provided variable training accuracy at each epoch. The accuracy value of the InceptionResNetV2 model starts at 0.9078 and fluctuates up to 0.9998 at epoch 20. Most deep retrieval techniques involve networks as local feature extractors that rely on models pretrained on large image classification datasets, such as ImageNet [46], and focus attention merely on developing image representations adequate for image retrieval on top of these features.

**Figure 7:**
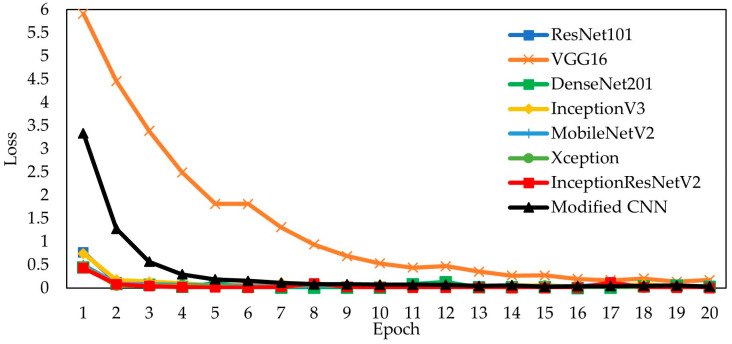
Visualization of the training loss on the pretrained and modified CNN models.

Figure 7 depicts the results of the training loss for the seven pretrained and proposed models. The loss value measures the network’s error. If the training accuracy increases steadily toward convergence, the loss value will converge to a value close to zero for good performance. The Xception model’s loss training data from the experimental results at epoch 20 has the lowest loss value of 0.0012, followed by InceptionResNetV2, ResNet101, InceptionV3, DenseNet201, modified CNN, MobileNetV2, and VGG16. As illustrated in Figure 6, the modified CNN model has a loss value that decreases steadily. The pretrained models exhibit overfitting. This is because the number of datasets is limited, and the ImageNet weight does not match the TenunIkatNet dataset. 

Table 1 compares the capacity and number of parameters of all models. The model’s size is also important, along with the accuracy and the loss rate. Small model sizes can be used in real-time applications [47]. The experimental findings demonstrate that MobileNetV2 [37] has the smallest model size and the smallest number of parameters, followed by VGG16 and the modified CNN. The MobileNetV2 model with the bottleneck residual block method can reduce the volume of data flowing into the network. The modified CNN model outperforms the MobileNetV2 and VGG16 models in terms of the training accuracy, loss value, and retrieval accuracy. The F1-score values are calculated for the proposed and pretrained models on the test dataset. The best F1-score value is 0.999 for the modified CNN model. The difference in the F1-score obtained by the ResNet101, DenseNet201, and InceptionV3 models was not significant. The lowest values are obtained by the VGG16 and MobileNetV2 models. 

#### 3.2.2. Image Retrieval Evaluation

**Figure 8:**
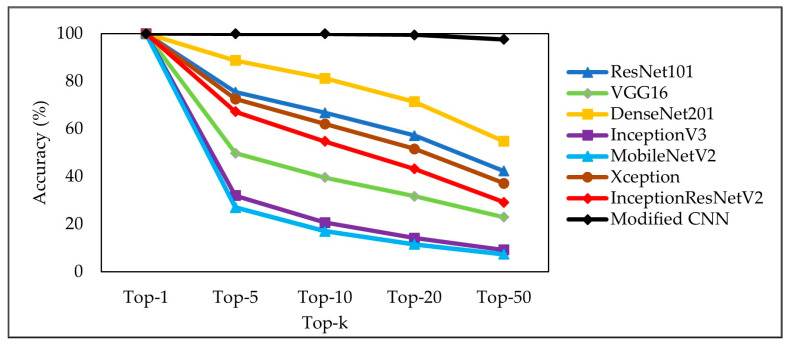
Retrieval accuracy of the pretrained and modified CNN models.

In these experiments, 960 testing images, 8 images from each category, were selected from the TenunIkatNet dataset. Figure 8 compares the retrieval accuracy of the pretrained CNN model and the proposed model. In top-1, all models obtain a 100% retrieval accuracy, which indicates that the same image as the request image is retrieved from the database. However, in top-5, top-10, top-20, and top-50, the retrieval accuracy obtained by the modified CNN model was the best, namely 99.96%, 99.88%, 99.50%, and 97.60%, respectively. The lowest retrieval accuracy was obtained by the MobileNetV2 model. The more images the model finds through the query process, the better the model’s performance. For example, for the top-20 results, that model should display 20 images related to the query image. If only 10 images are displayed correctly, the retrieval accuracy is 50%.

In general, the pattern cycle of the ikat woven fabric is highly complex and varied, so the association between the ikat woven fabric images is not effectively appraised, and this evaluation is necessarily subjective. Based on research [25], retrieval methods for printed fabrics with varying patterns have high error rates. Fabric images with complex textures are prone to mismatches. Therefore, for fabric images with similar colors, texture features can be considered to increase the retrieval accuracy [8]. Because the models were trained to achieve intraclass generalization, using off-the-shelf features from ImageNet-trained classification models may not be the best option for retrieval tasks [46]. Table 2 displays the retrieval error rates of multiple pretrained CNN and modified CNN models. The performance of the modified CNN models is significantly better than that of the pretrained models. 

Table 3 shows a comparison of the retrieval times. The LSH method also reduced the amount of time because of the binary code comparison between the two image vectors. It also emphasized reducing the vector dimension obtained from feature extraction based on probability. The hashing process for high-probability vectors was similar and grouped into the same category. The time parameter is also heavily affected by the state of the internet network and Google Collaboratory’s facilities. Based on the experimental results, the ResNet101 model provides the fastest retrieval time for each top-k. However, the difference in retrieval time with the proposed method is not significant.

**Figure 9:**
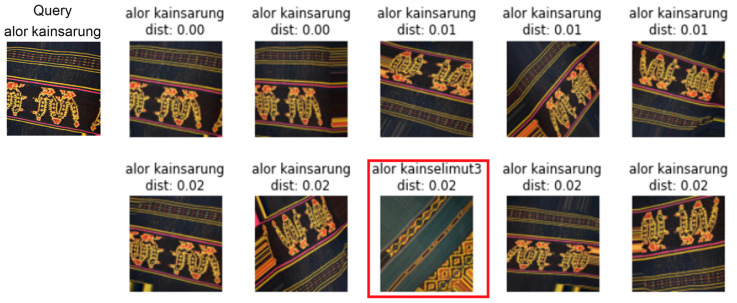
Visualization of the retrieval result of the DenseNet201 model for the top-10 images. The first column is the search image, and the subsequent columns are the obtained images. Incorrect images are identified by a red box. The type of ikat woven fabric is alor kainsarung.

**Figure 10:**
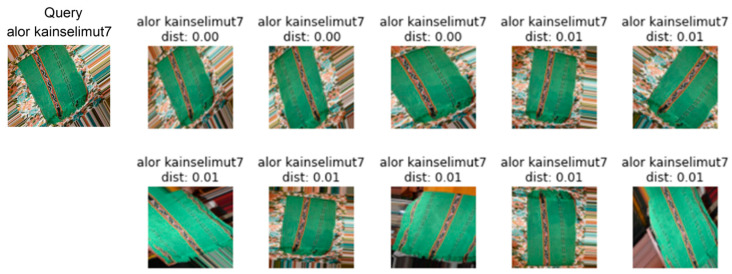
Visualization of the retrieval result of the modified CNN model for the top-10 images. The first column is the search image, and the subsequent columns are the recovered images. The type of ikat woven fabric is Alor Kainselimut7.

The error rate is employed to evaluate the ranking of the top-1, top-5, top-10, top-20, and top-50 images relative to the query image. Figure 9 and Figure 10 display the retrieval results for the top-10 images using DenseNet201 and the proposed models.

**Figure 11:**
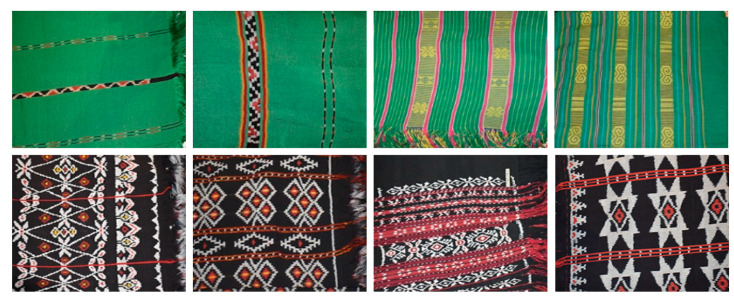
Examples of some similar images of ikat woven fabrics.

In most retrieval problems, the modified CNN model can obtain the same or related fabrics as the search image from the complex fabric images. The images with a texture similar to the requested image are displayed first [32]. Figure 11 shows several types of woven fabrics that have similar colors but different textures or shapes. Several types of woven fabrics result in low retrieval accuracy of the pretrained models. Moreover, in the modified CNN model, the accuracy did not decrease significantly. The modified CNN model with a few layers can maintain the essential features to distinguish the types of woven fabrics that are the same color but have different patterns or textures. Furthermore, a pretrained model with deep layers will result in the loss of basic features. Some pretrained models are unreliable because they poorly predict possible outputs for unknown inputs. One of the causes of poor machine learning performance is the color similarity between different types of woven fabrics. 

Based on the test results, the pretrained model performed better when applied to the retrieval of ikat woven fabrics. The CNN pretrained model performed better due to the characteristics of the ikat woven fabric, which is dominated by geometric shapes. The pretrained models trained on the ImageNet dataset are generally for image classification needs, so they may not be suitable for image retrieval [46]. In the initial convolution layer, the extracted image features were fundamental features of lines, edges, angles, and points so that the pretrained model with deep layers affected the feature map of the image of ikat woven fabrics.

When the modified CNN model is used as a feature extractor, it performs well. The characteristics of the TenunIkatNet dataset, which is dominated by geometric shapes, fit well with the modified CNN model. The number of convolution layers affects the initial extracted image from the convolution layer and affects the basic features of lines, edges, angles, and points. The visual feature detail components are obtained in the initial convolution layer. The deeper the number of convolution layers, the more basic features will be lost. This is shown by the test results of the pretrained model with a deeper convolution layer. The number of TenunIkatNet datasets is limited, but the modified CNN model experiences little overfitting. Future research will focus on increasing the number of TenunIkatNet datasets, reducing the model size and retrieval time, and increasing the retrieval accuracy.

Table 4 compares the results of the VGGNet model on the fabric and TenunIkatNet datasets. Previous research built large-scale fabric datasets, and the VGGNet model provided the highest accuracy [8]. This research used the VGGNet benchmark model with the TenunIkatNet dataset. The retrieval accuracies of the top-5 and top-20 are less than those of the state-of-the-art methods in previous studies. VGGNet models provide high accuracy on large fabric datasets. In [8] and this study, fabric image types with complex textures decrease accuracy. This shows that the texture features of fabric images are more dominant than the color features in the retrieval system. The modified CNN model is better than the VGGNet model on the TenunIkatNet dataset. The dataset’s characteristics and the model layer’s depth affect the retrieval accuracy. The number of datasets also affects the performance of the model.

## 4. Conclusions

This study collected images of Indonesian traditional woven fabric to form the TenunIkatNet dataset. The dataset is used as a knowledge base for artisans, trade promotions, and developing algorithms for image retrieval. The dataset consists of 120 classes and 4800 images. The images were also captured perpendicularly with the ikat woven fabric placed on different backgrounds, hung, and worn on the body, according to the utilization patterns. This research proposed a modified CNN (MCNN) model for image retrieval to provide a more accurate search for ikat woven fabrics. This model consists of three CL, MP, and FCL. Each CL applies 32 filters to obtain the map features. The filter size is 3 × 3 pixels, and the pooling layer uses a stride value of two. The dropout layer randomly deletes nodes in each iteration to reduce overfitting. The last FC layers are appended to combine the features detected from the image patches extracted by the previous layers and apply a linear transformation to the input vector through a weight matrix. Comparison experiments with ResNet101, VGG16, DenseNet201, InceptionV3, MobileNetV2, Xception, and InceptionResNetV2 show that the modified CNN model outperforms them in comprehensive retrieval performance. The research results show that the modified CNN performs well on the TenunIkatNet dataset with retrieval accuracies of 100%, 99.94%, 99.96%, 99.50%, and 97.60% for top-1, top-5, top-10, top-20, and top-50, respectively. The error rate results demonstrate that the modified CNN model performs better than the pretrained model.

## Figures and Tables

**Table 1:** Comparison of the model size, number of parameters, and F1-score.

Models	Model Size (MB)	Number of Parameters	F1-Score
ResNet101	491.2	42,904,056	0.998
VGG16	169.2	14,776,248	0.032
DenseNet201	213.1	18,552,504	0.998
InceptionV3	253.1	22,048,664	0.997
MobileNetV2	28.0	2,411,704	0.257
Xception	241.7	21,107,360	0.983
Inception ResNetV2	625.9	54,521,176	0.980
Modified CNN	170.9	14,927,672	0.999

**Table 2:** Retrieval error rates of the pretrained and modified CNN model.

Models	Retrieval Error Rate				
	*E*@*k* = 1	*E*@*k* = 5	*E*@*k* = 10	*E*@*k* = 20	*E*@*k* = 50
ResNet101	0	0.246	0.333	0.428	0.578
VGG16	0	0.502	0.605	0.683	0.771
DenseNet201	0	0.113	0.188	0.286	0.453
InceptionV3	0	0.680	0.793	0.858	0.908
MobileNetV2	0	0.730	0.830	0.885	0.926
Xception	0	0.275	0.380	0.485	0.629
Inception ResNetV2	0	0.328	0.452	0.569	0.708
Modified CNN	0	0.001	0.004	0.008	0.026

**Table 3:** Comparison of retrieval times.

Models	Retrieval Time (s)					Average Retrieval Time (s)
	Top-1	Top-5	Top-10	Top-20	Top-50	
ResNet101	0.1099	0.1006	0.0977	0.0973	0.0971	0.1005
VGG16	0.2225	0.2183	0.2171	0.2171	0.2166	0.2183
DenseNet201	0.3225	0.3202	0.3203	0.3199	0.3186	0.3203
InceptionV3	0.7749	0.7718	0.7712	0.7716	0.7718	0.7723
MobileNetV2	0.3810	0.3787	0.3783	0.3779	0.3779	0.3788
Xception	0.4404	0.4377	0.4366	0.4366	0.4362	0.4375
Inception ResNetV2	0.5089	0.5032	0.5036	0.5037	0.5036	0.5046
Modified CNN	0.1463	0.1423	0.1414	0.1415	0.1416	0.1426

**Table 4:** Performance comparison for top-1, top-5, and top-20.

Models	Accuracy (%)			
	Top-1	Top-5	Top-20	Average
VGGNet [8] (Fabric dataset)	91.14	98.43	99.88	96.48
VGGNet (TenunIkatNet)	100	49.79	31.70	59.83
Modified CNN (TenunIkatNet)	100	99.96	99.50	99.82

## Data Availability

Not applicable.
